# Evaluation of the Best Region for Measuring Eye Temperature in Dairy Cows Exposed to Heat Stress

**DOI:** 10.3389/fvets.2022.857777

**Published:** 2022-03-23

**Authors:** Hang Shu, Yongfeng Li, Tingting Fang, Mingjie Xing, Fuyu Sun, Xiaoyang Chen, Jérôme Bindelle, Wensheng Wang, Leifeng Guo

**Affiliations:** ^1^Agricultural Information Institute, Chinese Academy of Agricultural Sciences, Beijing, China; ^2^AgroBioChem/TERRA, Precision Livestock and Nutrition Unit, Gembloux Agro-Bio Tech, University of Liège, Gembloux, Belgium; ^3^Institute of Animal Science, Chinese Academy of Agricultural Sciences, Beijing, China

**Keywords:** dairy cows, heat stress, eye temperature, region of interest, infrared thermography

## Abstract

Eye temperature (ET) has long been used for predicting or indicating heat stress in dairy cows. However, the region of interest (ROI) and temperature parameter of the eye have not been standardized and various options were adopted by previous studies. The aim of this study was to determine the best ROI for measuring ET as the predictor of heat stress in dairy cows in consideration of repeatability and validity. The ET of 40 lactating Holstein dairy cows was measured using infrared thermography. The mean and maximum temperature of five ROIs—medial canthus (MC), lateral canthus, eyeball, whole eye (WE), and lacrimal sac (LS)—were manually captured. The results show that the ET of left eyes was slightly higher than that of right eyes. The ET taken in MC, WE, and LS within 2 min had a moderate to substantial repeatability. The maximum temperature obtained at the LS had the highest correlation coefficients with respiration rate and core body temperature (all *p* < 0.001). Therefore, the maximum temperature of LS should be considered by future studies that want to use ET as the predictor or indicator of heat stress in dairy cows.

## Introduction

Due to the advantages of non-invasive and non-contact measurement, infrared thermography (IRT) has been welcomed for measuring the welfare indicators and health status of livestock (1–3). Dairy cows are extremely vulnerable to heat stress due to their limited heat dissipation capacity and enormous heat production (4). However, the measurement of gold standard animal-based indicators of heat stress, i.e., respiration rate (RR) and core body temperature (CBT), is both time-consuming and labor-intensive (5). Therefore, body surface temperature measured using IRT has been used for predicting or indicating heat stress in dairy cows for a few years, among which the eye temperature (ET) has been considered most commonly due to its high correlation and agreement with CBT (6–8).

However, the region of interest (ROI) and temperature parameter of the eye have not been standardized and various options were adopted by previous studies. To name but a few, the mean or maximum temperature of the eyeball (EB) (9), the orbita plus surrounding (10), the entire eye (11), and the periocular and lacrimal caruncle (12) have been used for indicating heat stress in cattle.

To determine the best region to measure ET, two key parameters are of importance, i.e., repeatability and validity. The former focuses on whether measurements on the same subject under identical conditions over a short period of time are repeatable, whereas the latter focuses on the ability of a method to measure what it is intended to measure. For revealing heat stress of animals, validity is always expressed using the correlation with gold standard indicators (i.e., RR and CBT).

Some studies have used different methods to measure the repeatability of temperature readings of different ROIs of the cattle eye. Montanholi et al. (13) used the maximum temperature of the entire eye as the representative of ET, and correlation coefficients between two infrared images consecutively taken within 10 s were calculated to express repeatability. Byrne et al. (14) took 30 consecutive infrared images of the eye, the hoof, and the udder respectively, and repeatability was determined based on the level of precision that could be achieved by capturing 30 image replicates. Gloster et al. (15) also used the entire eye to obtain ET and the repeatability of thermal imaging was assessed by testing whether the difference in ET of two images taken within 10 min significantly differed from zero. However, none of the abovementioned studies was conducted under a heat stress condition, and only one ROI was adopted in each study. Therefore, it is interesting to know whether the temperature readings of different ROIs of the eye are repeatable across a heat stress event. As for the validity of ET in revealing heat stress in dairy cows, lots of studies have demonstrated a mild to strong correlation of ET with RR and CBT (10, 16, 17). Bleul et al. (17) reported that the maximum temperature of the entire eye, rather than the medial canthus (MC), had the highest correlation coefficient with the rectal temperature in 30 cows. However, a comprehensive evaluation of ROIs is still required.

The aim of this study was to determine the best ROI for measuring ET as the predictor of heat stress in dairy cows in consideration of repeatability and validity. Our hypothesis was that different ROIs would differ in their performance in reflecting heat stress in dairy cows.

## Materials and Methods

The experimental protocols were approved by the Experimental Animal Care and Committee of the Institute of Animal Science, Chinese Academy of Agricultural Sciences (approval number IAS2021-220).

### Experimental Location and Animals

The study was conducted at an organic intensive dairy farm in Shandong, China (coordinates: 34°50′37″N, 115°26′11″E; altitude: 52 m) from June to August in 2021. A total of 40 high-producing (daily milk yield: 40.0 ± 5.9 kg/day), primiparous and multiparous (parity: 2.6 ± 1.1), and mid-lactating (days in milk: 149.9 ± 18.2 days) Holstein dairy cows were randomly selected from a herd reared in a free-stall barn (15 m × 90 m). The barn was covered by a double-pitched roof and was oriented along the north-south longitudinal axis, and therefore, most of the solar radiation was prevented from reaching the cows inside the barn. The barn was equipped with a total of 20 fans (1.1 m in diameter; capacity: 25,000 m^3^/h each) and 40 sprinklers (1.5 L/min each; 1 min on and 4 min off) fixed 2.5 m above the ground. Fans were installed at the lying zone at an interval of 6 m and along the feeding line at an interval of 12 m, while sprinklers were installed along the feeding line at an interval of 2 m. Fans and sprinklers were operated normally during the entire study. Cows were milked three times per day at 08:30, 16:30, and 00:00 h. Cows were fed a total mixed ration three times per day after milking and had free access to clean water.

### Experimental Design

Physiological measurements were conducted twice on each test day (09:30–11:00 and 14:00–15:30 h). A veterinarian checked the health condition of the cows daily, and no cows were excluded due to health issues. The cows were resting quietly in a lying or standing posture during the measurements. Gold standard animal-based indicators (i.e., RR and CBT) were recorded by timing 15 flank movements (and converting to breaths/min) and using data loggers (DS1922L, Maxim Integrated, San Jose, CA, USA) attached to modified vaginal controlled internal drug releases (Pfizer Animal Health, New York, NY, USA), respectively.

The ET was recorded using a portable infrared camera (VarioCAM HR, InfraTec, Dresden, Germany) right after RR measurement. The camera had a spectral range from 7.5 to 14 μm, a temperature measuring range from −40 to 2,000°C, an accuracy of ± 2%, and a resolution of 640 × 480 pixels. The infrared images were taken twice within 2 min from each cow per measurement to evaluate the repeatability of measurements. All images were taken from the cows' side to capture eye regions with an angle of ~90° and a distance of ~1 m from the cows. To prevent the cows' body temperature from rising due to the stress caused by prolonged handling, we only measured the temperature of the eye that was on the side of the cow (i.e., left or right) that was close to the approaching thermographer for each cow per measurement.

Ambient temperature (Ta) and relative humidity (RH) were measured using a Kestrel 5400 heat stress tracker (Nielsen-Kellerman, Boothwyn, PA, USA). Temperature and humidity index (THI) was then calculated according to the following equation recommended by the National Research Council (18):


THI = (1.8 × Ta + 32) − (0.55 − 0.005 × RH) × (1.8 × Ta − 26)

### Infrared Image Processing

**Figure 1:**
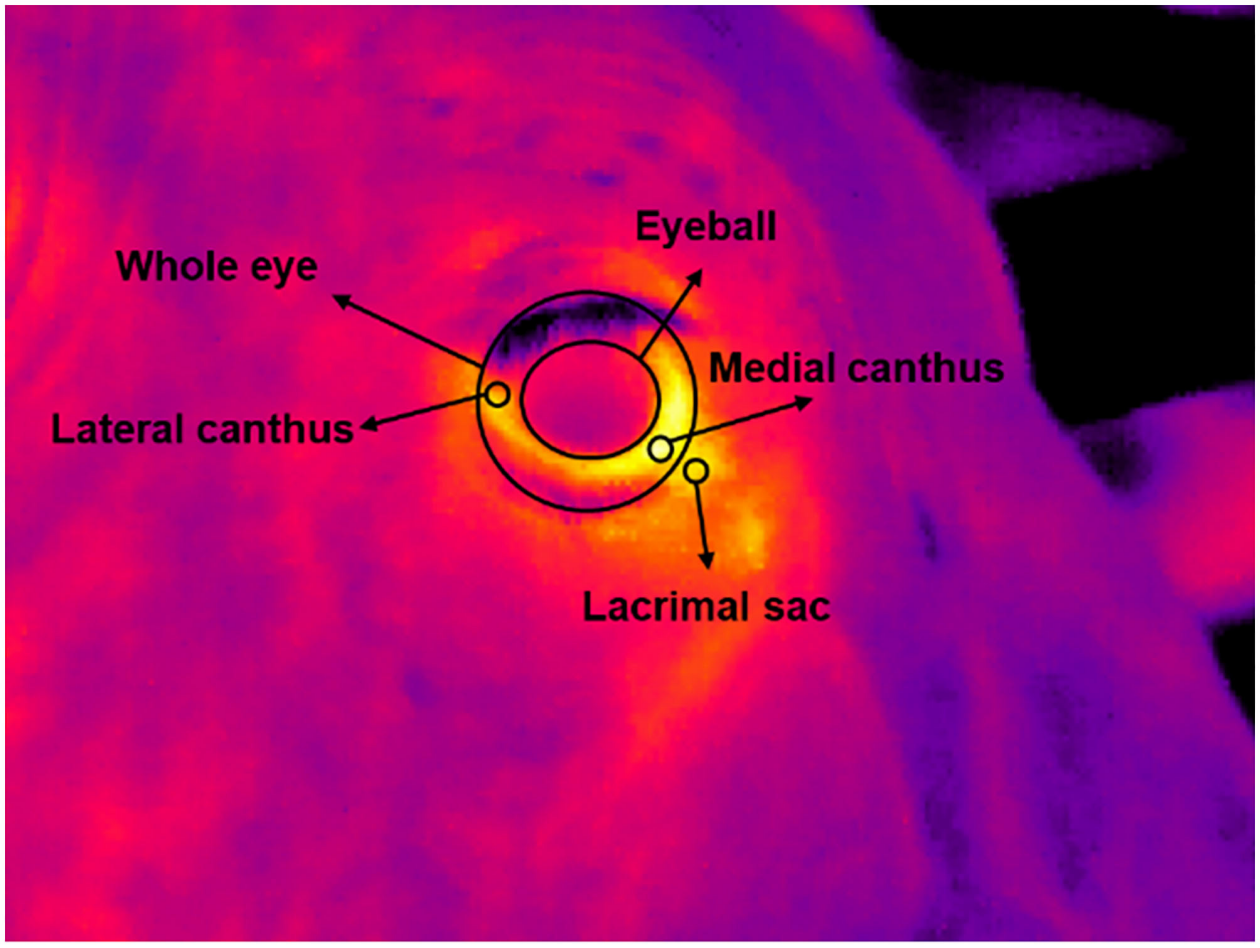
Five regions of interest of eyes for temperature measurement.

Infrared images were processed using IRBIS 3 Standard software (YSHY, Beijing, China). All images were calibrated by setting the emissivity to 0.98 and inputting the corresponding Ta record of each measurement. MC, lateral canthus (LC), EB, whole eye (WE), and lacrimal sac (LS) were manually located using appropriate circles to obtain the mean and maximum temperatures of the areas (Figure 1). Two replicated images that did not capture all five ROIs at the same time were manually eliminated. Consequently, 736 infrared images from 318 measurements were finally used for the following analyses.

### Statistical Analysis

All statistical analyses were performed using R version 4.1.0 (https://www.R-project.org/). Mean and maximum ET were analyzed separately by ROI and side, unless otherwise stated. To determine the temperature differences among ROIs and side (left and right), mean and maximum ET were analyzed separately using generalized linear mixed models with the “nlme” package including fixed effects of ROI, side, interaction between ROI and side, posture, random intercept effect of cow, and covariates of parity, days in milk, and THI. The repeatability of replicated ET taken within a 2-min interval was assessed by using the concordance correlation coefficient (CCC) for longitudinal repeated measures using the ccclon function included in the “cccrm” package with the index of observation ordered by time as the longitudinal unit for each cow. The correlations between ET and gold standard animal-based indicators were performed using the cor function. Significance was declared at *p* < 0.05.

## Results

### Descriptive Statistics

The descriptive statistics of meteorological and physiological variables are shown in Table 1. Among ROIs, the maximum temperature of WE (37.93°C) and MC (37.92°C) provided the highest temperature values, ~0.9°C lower than CBT (38.80°C). As expected, MC was a good representative of WE in terms of maximum temperature with nearly 80% of the images having maximum temperature located in MC. The coefficients of variation of all temperature variables (CBT and ET) lay between 1 and 2%, among which the mean temperature of EB and WE had the highest value of 1.79 and 1.86%, respectively. Besides, the coefficients of variation of maximum temperature in all five ROIs were lower than those of mean temperature.

**Table 1:** Number of observations (N), mean, standard deviation (SD), coefficient of variation (CV), minimum (Min), and maximum (Max) values of meteorological and physiological variables.

**Variable**	***N***	**Mean**	**SD**	**CV (%)**	**Min**	**Max**
THI	318	80.55	3.01	3.73	70.98	86.09
RR	318	70.70	19.93	28.19	27.17	145.16
CBT	168	38.80	0.45	1.17	38.00	40.10
MCmean	318	37.60	0.46	1.21	35.81	38.64
MCmax	318	37.92	0.41	1.08	36.74	38.96
LCmean	318	37.07	0.49	1.32	35.55	38.32
LCmax	318	37.43	0.45	1.19	36.13	38.63
EBmean	318	36.11	0.65	1.79	33.54	37.60
EBmax	318	37.34	0.50	1.34	35.96	38.50
WEmean	318	36.29	0.67	1.86	33.46	38.11
WEmax	318	37.93	0.41	1.07	36.86	38.97
LSmean	318	37.16	0.55	1.47	34.92	38.58
LSmax	318	37.42	0.51	1.36	35.72	38.81

*THI, temperature and humidity index; RR, respiration rate (breaths/min); CBT, core body temperature (°C); MC, medial canthus (°C); LC, lateral canthus (°C); EB, eyeball (°C); WE, whole eye (°C); LS, lacrimal sac (°C). Eye temperatures (°C) were summarized using the average measures of two replicated infrared images*.

### Temperature Differences Among ROIs and Sides

**Figure 2:**
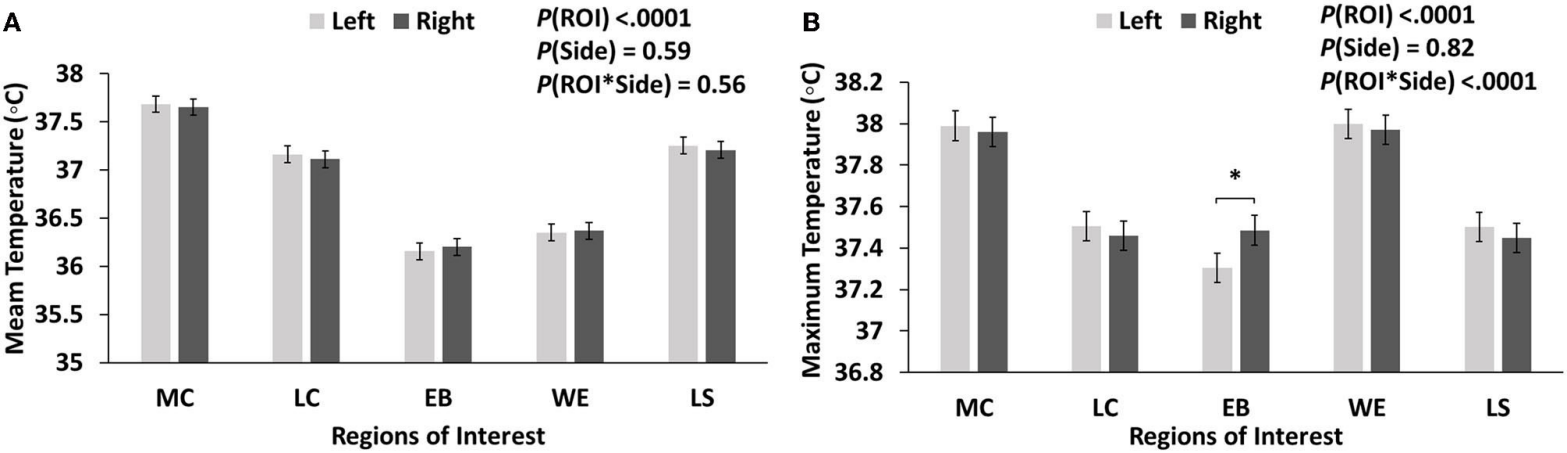
Mean **(A)** and maximum temperature **(B)** of left and right eyes obtained at five regions of interest (ROIs). Data are graphed using the least square mean ± standard error of the interaction (ROI by side). MC, medial canthus; LC, lateral canthus; EB, eyeball; WE, whole eye; LS, lacrimal sac.

For mean temperature, no significant difference between the left and right eyes was found for ET measured at all five ROIs (*P* > 0.05; Figure 2A). For maximum temperature, the EB temperature of left eyes (37.31°C) was significantly lower compared with that of right eyes (37.49°C) (*p* = 0.0007; Figure 2B). In general, a slightly higher ET (mean or maximum) was found in left eyes than right eyes except for EB (Figure 2).

### Imaging Repeatability

Considering left eyes and right eyes separately, five ROIs all yielded higher CCCs in their mean temperatures compared with maximum temperatures (Table 2). When comparing two eyes, left eyes always had CCCs higher than or equal to those of right eyes in both mean and maximum temperatures (Table 2). In general, MC, WE, and LS had CCCs higher than 0.90 for all combinations of temperature parameter (mean or maximum) and side (left or right) (Table 2), indicating that ET taken in these ROIs within 2 min had a moderate to substantial repeatability.

**Table 2:** Concordance correlation coefficients (95% confidence interval) of replicated infrared imaging measures (mean and maximum temperatures) taken from the left and right eyes, respectively.

**Side**	**Parameter**	**MC**	**LC**	**EB**	**WE**	**LS**
Left	Mean	0.96 (0.94, 0.97)	0.90 (0.86, 0.93)	0.94 (0.92, 0.96)	0.97 (0.95, 0.98)	0.95 (0.93, 0.97)
	Maximum	0.94 (0.92, 0.96)	0.85 (0.78, 0.90)	0.87 (0.81, 0.91)	0.94 (0.92, 0.96)	0.94 (0.91, 0.96)
Right	Mean	0.95 (0.92, 0.97)	0.85 (0.79, 0.89)	0.92 (0.90, 0.94)	0.95 (0.93, 0.97)	0.95 (0.92, 0.97)
	Maximum	0.92 (0.89, 0.95)	0.85 (0.79, 0.89)	0.72 (0.58, 0.82)	0.93 (0.90, 0.95)	0.92 (0.88, 0.95)

*MC, medial canthus; LC, lateral canthus; EB, eyeball; WE, whole eye; LS, lacrimal sac*.

### Correlations Between ET, RR, and CBT

The mean or maximum ET obtained at all ROIs on each or both sides were all significantly correlated with RR and CBT (all *p* < 0.001; Table 3). When taking left eyes and right eyes separately, the maximum temperature had higher or equal correlation coefficients with RR and CBT compared with the mean temperature in most ROIs of the eye except for LC in which the mean temperature was correlated more with RR (Table 3). The highest correlation coefficients of RR and CBT were both yielded from the maximum temperature obtained at the LS of left eyes (0.60 and 0.52, respectively; Table 3).

**Table 3:** Correlation coefficients of mean and maximum eye temperature (°C, left and/or right) with respiration rate (RR, breaths/min) and core body temperature (CBT, °C).

**Variable**	**Side**	**Parameter**	**MC**	**LC**	**EB**	**WE**	**LS**
RR	Left eye	Mean	0.48	0.48	0.36	0.37	0.58
		Maximum	0.49	0.45	0.39	0.49	0.60
	Right eye	Mean	0.44	0.41	0.33	0.35	0.50
		Maximum	0.44	0.39	0.35	0.44	0.51
	Two eyes	Mean	0.46	0.45	0.35	0.36	0.54
		Maximum	0.47	0.42	0.37	0.46	0.55
CBT	Left eye	Mean	0.38	0.37	0.25	0.25	0.48
		Maximum	0.43	0.39	0.34	0.42	0.52
	Right eye	Mean	0.48	0.36	0.31	0.38	0.50
		Maximum	0.48	0.38	0.38	0.48	0.50
	Two eyes	Mean	0.43	0.37	0.28	0.32	0.49
		Maximum	0.45	0.38	0.34	0.45	0.51

*MC, medial canthus; LC, lateral canthus; EB, eyeball; WE, whole eye; LS, lacrimal sac. All p < 0.001*.

When combining both eyes into analysis, the maximum temperature had better correlation coefficients with RR and CBT compared with the mean temperature in most ROIs of the eye except for the LC in which the mean temperature still correlated more with RR (Table 3). Besides, the correlation coefficients were pooled to some extent, with the results lower than those from left eyes but higher than those from right eyes (Table 3). As expected, the maximum temperature obtained at the LS of both eyes still correlated most with RR and CBT (*r* = 0.55 and 0.51, respectively; Table 3).

## Discussion

According to the revised heat stress categories proposed by Collier et al. (19), the cows were exposed to mild to severe heat stress. Since this study lasted 3 months in the summer, the effects of long-term heat stress were well captured. In the present study, five ROIs of the eye were all determined using images taken from the side of the cows' faces. Some studies also captured ET using the front area of the cattle face (20, 21). However, front images were not considered in the present study since a minor variation in the angle of the infrared camera to the target could result in a very different temperature value. To the best of our knowledge, this is the first study to comprehensively compare the temperatures among various ROIs of the cow's eye.

The temperature of the left and right eyes of cows is rarely compared. Most of the previous studies did not distinguish between the left and right eyes when obtaining ET. Our results show that the left eye seemed to have a higher temperature, better repeatability, and better correlation with gold standard animal-based indicators. Besides, the combined dataset of both left and right eyes provided a pooled correlation with RR and CBT, which was better than right eyes solely and worse than left eyes solely. Byrne et al. (14) also found that left eyes were more repeatable than right eyes, where 70.20% of the total variation could be explained by cow. However, left eyes were found 0.18 and 0.53°C lower than right eyes in the maximum and mean temperature, respectively, which is opposite to our results. A higher temperature in the left eye than the right eye was also found by the study of Church et al. (22) where 79 Holstein dairy cows were measured ET using IRT. The cows were raised in a barn oriented along the north-south longitudinal axis, and an adjustable perforated awning was used to provide three treatment conditions: direct sunlight (left eyes), indirect sunlight (right eyes), and shaded (both eyes). Even under the shaded condition, a 0.14°C higher temperature was found in left eyes than in right eyes. Rogers (23) reported that both acute and chronic stress of an animal are processed by the right hemisphere of the brain. Since the right hemisphere of the brain is linked to the left eye (24), it may explain why our results show that the temperature of the left eye was better at indicating heat stress in dairy cows. Due to the fact that rumen is on the left side of the cow's body, Brcko et al. (11) measured ET only from the cows' right side to prevent the impact of digestive processes. However, our results might indicate a negligible effect of rumination and digestion on the temperature of the left eye since the left eye and the rumen are anatomically far apart. It is also worth noting that the wind direction along the feeding line was from left to right for cows that were eating in the present study. Thus, we speculate that the better performance of the left eye might also be partly attributed to the direct exposure to fans. Indeed, evaporative cooling including sprinklers and fans together has shown a significant effect on reducing the body surface temperature of cows by promoting local cutaneous evaporation (25, 26). However, further studies are required in which the direction of the air flow relative to the cows is strictly controlled.

As expected, the mean temperature of the ROIs always had better repeatability compared with the maximum temperature, which is consistent with the study of George et al. (27). However, maximum temperature provided better correlations with RR and CBT in most ROIs, reconfirming that maximum temperature could better reflect cows' thermoregulation under heat stress (28). Also, Uddin et al. (29) reported that the maximum temperature of the eye was more correlated with stress- and productivity-related parameters than the mean temperature. Among five candidate ROIs of the eye, LS was determined to be the best area for ET measurement using IRT in dairy cows due to the consistently good results in repeatability and correlations with RR and CBT. Besides, MC and WE had similar results when using maximum temperature since nearly 80% of the images had a maximum temperature located in the MC area. It is well known that the lacrimal caruncle and the small area around the medial posterior palpebral border of the lower eyelid have abundant capillaries of the maxillary and infraorbital arteries, which are innervated by the sympathetic system (8, 30). When the animals are exposed to heat stress, vasodilation leads to an increased surface temperature of these areas to promote heat dissipation (4). On the other hand, EB was found to have a relatively lower CCC for replicated infrared imaging measures and a lower correlation with RR and CBT compared with other ROIs. This may be due to inaccurate temperature readings caused by lacrimal secretions during heat stress. Vasodilation increases blood flow, thereby promoting the secretion of lacrimal gland fluid (31). Although poor quality images with obvious tears or dirt obscuring the ROIs have been manually eliminated, some invisible secretions might cover the surface of the EB and affect IRT by reflecting most infrared light, resulting in the lower temperature value, which was actually the temperature of lacrimal secretions. The higher coefficient of variation (1.79%) and the lowest minimum temperature (33.54°C) of the mean temperature of EB also supported the presence of secretions to have biased the actual temperature of EB downwards.

One of the limitations of the present study is that the size of ROIs was not constrained among infrared images. This could have an impact on the result of mean temperature. However, since the distance between the camera and the cows could not be fixed to 1 m completely, it was difficult to use fixed pixel sizes for ROIs. Besides, the maximum temperature was less likely to be influenced by slightly changed pixel sizes and its result should remain robust under our study design. On the other hand, the average temperature of the left and right eyes was used in previous studies (27). However, to prevent the cows' body temperature from rising due to the stress and anxiety caused by prolonged handling, we only measured the temperature of one eye from each cow per measurement. Thus, the average temperature of the left and right eyes was not able to be evaluated in this study. Furthermore, only the infrared images with all five ROIs available were selected for analysis; however, the actual situation is likely to be less favorable. For example, partially closed eyes and eyes covered by secretions or dirt are very common in the field, and will result in difficulty to obtain ROIs. Thus, we suggest that the maximum temperature of LS, MC, or WE should be used as representative ET when available.

## Conclusion

Collectively, this study demonstrates that the most commonly used ROIs (WE and MC) in the previous studies provided acceptable performance in reflecting the thermoregulatory response of dairy cows exposed to heat stress, whereas LS was the best area to obtain ET. More interestingly, left eyes were found to have a higher correlation with gold standard animal-based indicators (i.e., RR and CBT). Further studies are required to evaluate this phenomenon in which the direction of the airflow relative to the cows is strictly controlled.

## Data Availability Statement

The original contributions presented in the study are included in the article/supplementary material, further inquiries can be directed to the corresponding author/s.

## Ethics Statement

The animal study was reviewed and approved by the Experimental Animal Care and Committee of Institute of Animal Science, Chinese Academy of Agricultural Sciences (approval number IAS2021-220).

## Author Contributions

HS and JB: experiment design. HS, YL, TF, MX, FS, and XC: experiment execution. HS: data processing and analysis and writing—original draft. JB: writing—review and editing. WW and LG: resources and supervision. All authors approved the final manuscript.

## Funding

This study was financed by the Major Science and Technology Program of Inner Mongolia Autonomous Region, Grant Number 2020ZD0004, the Key Research and Development Program of Hebei Province, Grant Number 20327202D, and the Key Research and Development Program of Hebei Province, Grant Number 19220119D.

## Conflict of Interest

The authors declare that the research was conducted in the absence of any commercial or financial relationships that could be construed as a potential conflict of interest.

## Publisher's Note

All claims expressed in this article are solely those of the authors and do not necessarily represent those of their affiliated organizations, or those of the publisher, the editors and the reviewers. Any product that may be evaluated in this article, or claim that may be made by its manufacturer, is not guaranteed or endorsed by the publisher.
